# Dedifferentiation: inspiration for devising engineering strategies for regenerative medicine

**DOI:** 10.1038/s41536-020-00099-8

**Published:** 2020-07-31

**Authors:** Yongchang Yao, Chunming Wang

**Affiliations:** 1 Department of Joint Surgery, The First Affiliated Hospital of Guangzhou Medical University, 510120 Guangzhou, China; 2 Guangdong Key Laboratory of Orthopaedic Technology and Implant Materials, Guangzhou, China; 3 State Key Laboratory of Quality Research in Chinese Medicine, Institute of Chinese Medical Sciences, University of Macau, Macau SAR, China

**Keywords:** Tissue engineering, Regenerative medicine

## Abstract

Cell dedifferentiation is the process by which cells grow reversely from a partially or terminally differentiated stage to a less differentiated stage within their own lineage. This extraordinary phenomenon, observed in many physiological processes, inspires the possibility of developing new therapeutic approaches to regenerate damaged tissue and organs. Meanwhile, studies also indicate that dedifferentiation can cause pathological changes. In this review, we compile the literature describing recent advances in research on dedifferentiation, with an emphasis on tissue-specific findings, cellular mechanisms, and potential therapeutic applications from an engineering perspective. A critical understanding of such knowledge may provide fresh insights for designing new therapeutic strategies for regenerative medicine based on the principle of cell dedifferentiation.

## Introduction

Regeneration has always been a dream of humans, as shown in various fairy tales. Some invertebrates and amphibians have extraordinary capacity for regeneration. Zebrafish are able to fully regenerate their hearts, even after 20% of the ventricle is removed^1^. Earthworms readily reform two new duplicates after being cut in half^2^. Some amphibians, such as newts, create new limbs, tails and jaws with identical structures or functions to the lost body parts after amputation^3^, which is regarded as complete regeneration. However, at the organismal level, humans (like many other mammals) have a limited capacity for regeneration. The structure or functions of a damaged organ may be repaired to a compromised extent compared to the native status, and such “incomplete” regeneration usually occurs within this specific organ^4^. Typical cases include scarring in the skin that restores coverage without perspiration, as well as fibrocartilage that provides a structure different from articulate cartilage (and therefore inferior functions). Thus, these complete regenerative phenomena are prompting researchers to determine the underlying mechanisms and transform this mythological image into reality for humans. Dedifferentiation is regarded as one of the mechanisms involved in regeneration, as it enables cells, especially those without proliferative potential, to proliferate again and redifferentiate, leading to the replacement of the lost cells.

There are a variety of cell types throughout multicellular organisms. All of these cells originate from a simple zygote through a series of processes such as cell division and differentiation^5^. During differentiation, the fundamental basis of development of an organism, a less specialized cell type gradually transforms into a more specialized cell type, which is constrained to a stable morphology, structure and function. Moreover, the process of differentiation reduces the self-renewal ability and pluripotency of the cell. Differentiation continues during adulthood to maintain homeostasis. Dedifferentiation is a cellular process by which cells grow in reverse, from a partially or terminally differentiated stage to a less differentiated stage within their own lineage. In general, the phenomenon is manifested by a change in the shape, gene expression pattern, protein expression pattern and function. For example, dedifferentiated chondrocytes experience a change in phenotype after repeated culture in monolayers. The phenotype of spherical chondrocytes changes to spindle-shaped fibroblastic-like cells. Meanwhile, dedifferentiation results in a shift in the expression of the type II collagen (Col II) gene to type I collagen (Col I) and thus a corresponding change in the production of Col II protein to Col I protein in the extracellular matrix (ECM)^6^. In addition, these changes also lead to a switch from hyaline cartilage to fibrous cartilage, which would considerably change the function of articular cartilage^7^.

In recent decades, an increasing number of researchers have proposed that the induction of dedifferentiation shows promise to repair injured tissue in the clinic^4,8^. Terminally differentiated cells obtained in a noninvasive manner are potentially transformed to pluripotent stem cells through dedifferentiation. Then, these cells would be propagated in vitro, differentiated into objective cells and transplanted in vivo to achieve the regeneration of the damaged organ. Based on accumulating evidence, signaling pathways play a critical role in the process of dedifferentiation. Activation of the Wnt/β-catenin signaling pathway induces the dedifferentiation of epidermal cells^9^, articular chondrocytes^10^, or endothelial cells^11^ for regeneration. In addition, some specific biomolecules trigger the dedifferentiation of vascular smooth muscle cells^12^ or chondrocytes^13^ via the mitogen-activated protein kinase (MAPK) pathways. Also, innovative biomaterials are designing and creating to regulate dedifferentiation^14,15^. As our understanding of the molecular mechanisms of dedifferentiation improves, therapeutic methods using dedifferentiation present tremendous potential.

In this review, we compile the literature on recent advances in research of dedifferentiation, with an emphasis on the current knowledge of dedifferentiation processes, possible mechanisms and the therapeutic applications with an engineering perspective. A comprehensive understanding of such knowledge may provide fresh insights into the potential use of dedifferentiation in translational science.

### Dedifferentiation in tissue regeneration (as a physiological mechanism)

The phenomena of dedifferentiation exists in various tissues and organs from plants, amphibians and animals, manifesting mainly as re-entry into the cell cycle, the acquisition of a stem cell-like phenotype, the expression of stem cell markers and redifferentiation to regenerate damaged tissues^16^. Here, the dedifferentiation of mammalian cardiomyocytes and neurons, which have been studied extensively, will be discussed in detail.

#### Dedifferentiation of cardiomyocytes

**Fig. 1:**
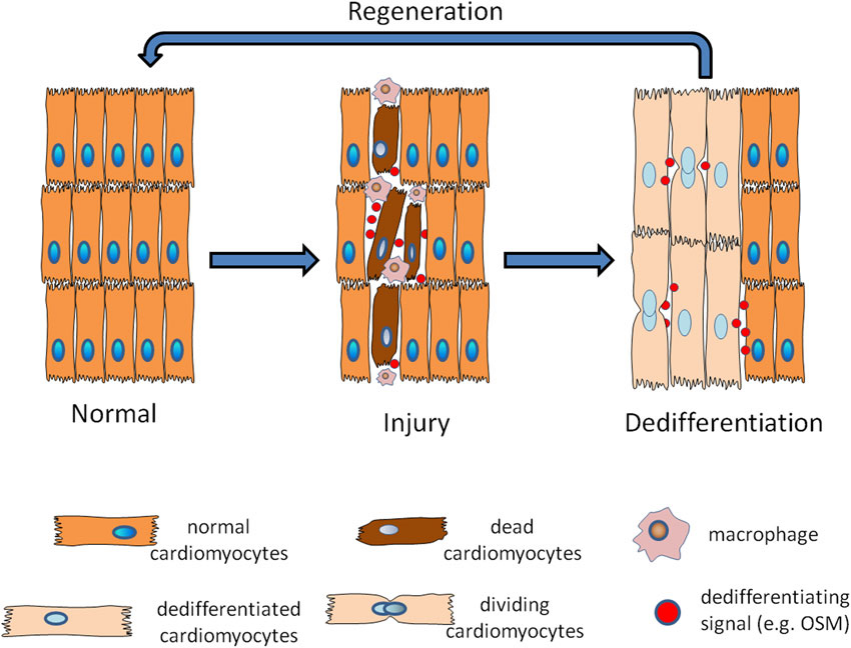
Schematic showing an overview of cardiomyocyte dedifferentiation during cardiac regeneration. Macrophages infiltrate the damaged heart to remove debris and release OSM to induce the dedifferentiation of surviving cardiomyocytes. Then, cardiomyocytes switch from the expression pattern of mature cardiomyocytes to a more immature state, enabling the cells to survive under unfavorable conditions, enter the cell cycle to proliferate, re-establish cell–cell contacts and recover their functions.

Mammalian cardiomyocytes stop multiplying soon after birth, which leads to the inability to replace the heart muscle cells that are damaged by myocardial infarction and other illnesses^17^. However, Porrello et al.^18^ observed a transient regenerative potential of the neonatal murine heart induced by the dedifferentiation of cardiomyocytes. Moreover, cardiomyocyte remodeling, which includes the restructuring of the normally existing structures and rearrangement of the cytoskeleton, is vital for the normal function of heart^19^. A recently proposed hypothesis states that cardiomyocyte remodeling is closely related to dedifferentiation, which is characterized by a switch in the expression pattern of mature cardiomyocytes to a more immature state, enabling cardiomyocytes to survive under unfavorable conditions, enter the cell cycle to proliferate and recover their functions^20,21^. MacLellan’s group demonstrated in mouse model that dedifferentiation may be an essential prerequisite for cardiomyocyte proliferation^22^. Both early genes, such as α-smooth muscle actin (α-SMA) and GATA4, and characteristic markers that are generally expressed in stem cells, such as Runx1 and Dab2, are re-expressed in dedifferentiated cardiomyocytes, which might facilitate proliferation and improve the multipotent differentiation capacity^22,23^. The dedifferentiation of cardiomyocytes may be an alternative pathway to induce regeneration, as observed in the newt, whose heart is regenerated through the dedifferentiation of mature cardiomyocytes and subsequent proliferation (Fig. 1). The corresponding mechanisms have not been completely elucidated. Numerous studies have been performed to provide insights into the underlying mechanisms.

An in vitro model in which adult rabbit cardiomyocytes were cocultured with cardiac fibroblasts was established^24^. The model exhibits similar morphological changes to dedifferentiating cardiomyocytes in vivo, and thus may be a useful tool to investigate the factors and the pathways involved in dedifferentiation in vivo. Recently, Kubin et al.^25^ reported a key role for the inflammatory cytokine oncostatin M (OSM), a member of the interleukin-6 family, in the mechanism regulating cardiomyocyte dedifferentiation. OSM triggers the Ras/MEK/Erk cascade to induce dedifferentiation of in vitro rat cardiomyocyte via OSM receptor β. A positive feedback loop mediates murine cardiomyocyte dedifferentiation; it comprises Yes-associated protein (YAP), transcriptional enhancer factor (TEAD1) and OSM—where the YAP-TEAD1 pathway stimulates OSM expression, OSM activates YAP-TEAD1, and OSM also upregulates OSM receptors through the YAP-TEAD1 pathway^26^. The size of OSM-treated cardiomyocytes also increases, which may allow them to re-establish cellular contacts to promote cellular survival^27^.

In addition, during the process of limb regeneration in urodele amphibians, the retinoblastoma (RB) protein has a very important role in facilitating the re-entry of differentiated cells into the cell cycle. During this process, mature cells dedifferentiate through the inactivation of RB and then enter the cell cycle^28^. The findings from numerous studies on the role of RB are consistent with this finding. The inactivation of RB, which is attributed to phosphorylation by increased numbers of cyclin D1/cdk4 complexes, is related to cardiomyocyte hypertrophy in congestive heart failure^29^. According to MacLellan et al.^30^, mice lacking RB and RB-like 2 have a larger heart and exhibit increased cardiomyocytes proliferation. Zaglia et al.^31^ were the first researchers to report that GATA4, a zinc-finger transcription factor, is expressed by fibroblasts in the adult rat heart, but not in fibroblasts from the gut, skin and skeletal muscle. In addition, the authors established an in vitro coculture experiment with cardiac fibroblasts and cardiomyocytes. Cardiac fibroblasts enhance the dedifferentiation of adult cardiomyocytes, a change that is strongly correlated with cell cycle reprogramming, as evidenced by the expression of DNA synthesis markers. Stimulation of GATA4 by overexpression of GRP78 (glucose-regulated protein of 78 kDa) can promote cardiomyocyte growth in mice^32^. However, further studies are needed to investigate the underlying mechanisms by which GATA4 affects cell division.

#### Dedifferentiation of neurons

**Fig. 2:**
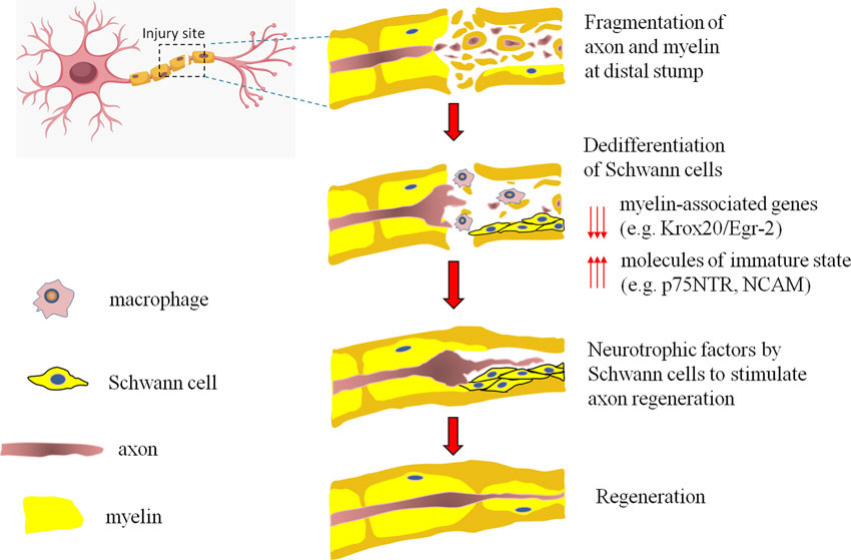
Schematic showing an overview of nerve regeneration after injury induced by the dedifferentiation of Schwann cells. The fragmentation of axons and myelin occurs at the injury site. The mature myelinating Schwann cells dedifferentiate, regain the expression pattern associated with immature Schwann cells and proliferate. Then, Schwann cells remove myelin and axonal debris after dedifferentiation by themselves or by recruiting circulating macrophages and produce neurotrophic factors to support axon regeneration.

Following nerve injury, Schwann cells regenerate. The mature myelinating Schwann cells dedifferentiate, regain the expression pattern associated with immature Schwann cells, proliferate and subsequently redifferentiate to induce nerve repair^33^. Schwann cells remove myelin and axonal debris after dedifferentiation by themselves or by recruiting circulating macrophages^34^. Upon dedifferentiation, Schwann cells downregulate myelin-associated genes that are essential for the myelination process, such as myelin protein zero and Krox20/Egr-2^35^, and re-express molecules associated with immature states, such as p75 neurotrophin receptor (p75NTR), neural cell adhesion molecule (NCAM) and L1^36^. Moreover, Schwann cells produce neurotrophic factors to support axon regeneration^37^ (Fig. 2).

Based on accumulating evidence, several intracellular signaling pathways are involved in Schwann cell dedifferentiation. Notch signaling negatively regulates myelination, accelerates the dedifferentiation of mature Schwann cells and promotes the proliferation of immature Schwann cells^38^. A series of studies has focused on mitogen-activated protein kinase (MAPK) pathways in Schwann cell dedifferentiation. Although extracellular signal-regulated kinase (ERK) signaling is implicated in Schwann cell dedifferentiation^39^, increased ERK phosphorylation alone is not sufficient to cause dedifferentiation without colony-stimulating factor (CSF)-1-activated macrophage reactions^40^. The transcription factor c-Jun, which is the terminal component of the Jun-N-terminal kinase (JNK) pathway, is a negative regulator of myelination^41^. Parkinson et al.^42^ genetically depleted c-Jun from murine Schwann cells and observed a delay in demyelination after injury and the suppression of dedifferentiation. Recently, the transient reactivation of mTOR complex 1 was shown to be necessary to increase c-Jun translation and Schwann cell dedifferentiation after mice nerve injury^43^. Additionally, c-Jun contributes to the production of neurotrophic factors, including glial cell line-derived neurotrophic factor (GDNF) and Artemin, which is beneficial for axonal regeneration^44,45^. The activation of p38 MAPK induces c-Jun expression and drives rat Schwann cell dedifferentiation^46^. Park and colleagues^47^ reported an important role for the Rac1 GTPase (Rac) in Schwann cell dedifferentiation. Rac controls the demyelination of murine Schwann cells in Schmidt-Lantermann incisures and induces c-Jun expression in injured sciatic nerves. Rac also regulates the expression of GDNF and p75NTR, which are related to regeneration. Furthermore, the neuregulin-Rac-MKK7-JNK/c-Jun pathway is required for Schwann cell dedifferentiation following rat nerve injury^48^. As shown in the study by Jung’s group, extracellular adenosine triphosphate inhibits rat Schwann cell proliferation and Schwann cell dedifferentiation by blocking the lysosomal activation^49^. Likewise, increased cAMP levels induce and maintain the differentiation of Schwann cells, while the removal of cAMP rapidly induces the dedifferentiation and proliferation of Schwann cells^42,50^. Moreover, according to Viader et al.^51^, miRNAs expressed in Schwann cells play a key role in modulating murine Schwann cell response to nerve injury. The authors identified interactions of miR-34a with Notch1 and Ccnd1 to promote the dedifferentiation and proliferation of Schwann cells, and miR-140 influenced the remyelination.

In addition, astrocytes, which are widely distributed throughout the central nervous system (CNS), dedifferentiate into neural stem/progenitor cells (NSPCs) upon exposure to appropriate stimuli based on compelling evidence^52,53^. Conditioned medium from scratch-insulted astrocytes (ACM) induces astrocytes to regain NSPCs potentials and these NSPCs of astrocytic origin redifferentiated into neuronal and glial cells^54,55^. Overexpression of Ezh2 increases the expression of neural stem cell genes and downregulates the expression of astrocytic genes, but these changes were insufficient to induce a complete dedifferentiation^56^. A series of studies has been conducted by Yang et al.^57^ to investigate the underlying mechanisms involved in the astrocyte dedifferentiation process. Using an in vitro scratch-wound model, the authors revealed that ACM treatment stimulated the rat astrocytes to dedifferentiate into radial glial progenitor cells with the characteristic radial glial-like morphology, re-expression of nestin, RC2 and paired homeobox proteins, and increased multipotent differentiation capability. Concomitantly, the progressive increase in the level of ErbB2 protein level suggested that the activation of the ErbB2 signaling pathways may lead to all these phenotypes in astrocytes. Furthermore, fibroblast growth factor 4 (FGF4), which is actively involved in neurogenesis^58^, is required for the astrocyte dedifferentiation because it activates the PI3K/Akt/p21 signaling pathway^59^. Astrocytes lacking FGF4 do not undergo dedifferentiation, while the application of FGF4 alone to astrocyte cultures fails to elicit the reprogramming of astrocytes towards NSPCs. In addition, in vitro experiments confirmed that the increase in sonic hedgehog (Shh) production after invasive injury plays a key role in rat astrocyte dedifferentiation^60^. The authors observed a significant inhibition of astrocyte dedifferentiation into NSPCs using a neutralizing antibody to inhibit Shh. Moreover, the addition of Shh alone had little effect on the dedifferentiation process and reversion to NSPCs. Based on these results, Shh is necessary but not sufficient to induce astrocyte dedifferentiation. However, in a subsequent study, Sirko drew a distinct conclusion that Shh signaling is necessary and sufficient to promote the proliferation of astrocytes in a mouse model and trigger their ability to form neurospheres in vitro^61^. More recent studies further demonstrate that Shh signaling effectively promotes conversion of murine astrocytes to a pluripotent state^62,63^. The treatment with two chemically distinct agonists of the Shh signaling promoted the elongation and proliferation of murine astrocytes^64^. The mechanism was related to the crosstalk between Sox2/Shh-targeted downstream signals and PI3K/Cdk2/Smurf2 signaling^65^.

### Dedifferentiation in diseases (as a pathological factor)

As mentioned above, the induction of dedifferentiation might be beneficial for tissue recovery in various organs. However, it might also cause some pathological events in certain tissues. In this case, cartilage is one of the classic tissue models and will be discussed in details.

Chondrocyte dedifferentiation has some similarities to osteoarthritis, such as a significant decrease in the expressions of aggrecan and Col II^66^. Chondrocyte dedifferentiation induces substantial changes in the levels of cell surface markers and cytoskeletal proteins, as well as the appearance of nonspecific ECM, which would ultimately lead to osteoarthritis. In addition to changes in the aggrecan to versican and Col II to Col I ratios and the deposition of collagen type X^67^, which are markers of the chondrocyte dedifferentiation status, the ratio of the cell surface markers CD14 to CD90 at the mRNA and protein levels has been corroborated as a new cell membrane-based differentiation index^68^. In addition, CD166 is an indicator of chondrocyte redifferentiation^69^. As shown in the study by Hong and Reddi, microRNA-221 and microRNA-222 are upregulated during dedifferentiation, while microRNA-140, microRNA-143 and microRNA-145 are downregulated^70^. Moreover, chondrocyte dedifferentiation controls the mechanical properties of the cell, such as increasing cell stiffness by enhancing membrane-actin adhesion^71^. However, chondrocyte dedifferentiation is not related to cell division^72^.

An increasing number of studies have been conducted to explore the cytokines and signaling pathways that are involved in chondrocyte dedifferentiation. MAPK signaling, consisting of ERK, JNK and p38, has very important roles in chondrocyte phenotype and metabolism. Rosenzweig et al.^73^ investigated the role of MAPK signaling in the dedifferentiation of in vitro primary bovine chondrocyte using inhibitors to block ERK, JNK, and p38. The inhibition of ERK and JNK by their respective inhibitors PD98059 and SP600125 significantly increased the expression of both chondrogenic marker genes and fibrotic genes, while blockade of p38 with SB203580 significantly upregulated Col II expression but inhibited Col I expression. Thus, p38 has a substantial contribution to the dedifferentiation of primary chondrocytes under normal monolayer culture conditions. Interestingly, Kim and colleagues^74^ reported that cytokine-induced apoptosis inhibitor-1 (CIAPIN-1) induced the dedifferentiation of in vitro cultured rabbit articular chondrocytes by activating ERK-1/2 and inactivating p38. The inhibition of ERK-1/2 by PD98059 appears to suppress the CIAPIN-1-induced dedifferentiation, while the addition of SB203580 to inhibit p38 promotes dedifferentiation. This inconsistency is possibly because—(i) other signaling molecules activated and (ii) the extent of p38 inhibition. The latter could be more likely because this group further observed that p38 inhibition under both CIAPIN-1 promoter and inhibitor SB203580 enhanced dedifferentiation. Two more subsequent findings by this group^75,76^ are similar, both involving a pre-inducing factor. Kim and colleagues also performed a series of in vitro studies in this area. They investigated the effects of thymoquinone (TQ) and PEP-1-SIRT2 on the dedifferentiation and inflammation of rabbit chondrocytes^13,77^. TQ or PEP-1-SIRT2 induce the expression of cyclooxygenase-2 (COX-2), causing the dedifferentiation of rabbit articular chondrocytes^78^ and subsequently leading to the dedifferentiation of chondrocytes through the ERK pathways. TQ-induced inflammation is mediated by the phosphoinositide 3-kinase (PI3K) and p38 pathways, while PEP-1-SIRT2-induced inflammation is mediated by the p38 and ERK pathways. The authors also reported that salinomycin (SAL) causes the dedifferentiation of rabbit articular chondrocytes via the ERK pathway, as determined by increased ERK activity without alterations in JNK and p38 activity following treatment with SAL^79^. Recently, the authors revealed that PEP-1-glutaredoxin-1 increased endoplasmic reticulum-stress and then stimulated the dedifferentiation of rabbit chondrocytes via the ERK-1/2 pathway^75^. Another Korean lab clarified the molecular mechanisms by which interleukin (IL)-1β, a proinflammatory cytokine associated with various cellular activities, induced the dedifferentiation of in vitro cultured primary rabbit chondrocytes^80^. IL-1βinduces the expression of nicotinamide phosphoribosyltransferase (NAMPT) and then activates SIRT1, which subsequently induces the activation of ERK and p38. In turn, ERK and p38 activate SIRT1, forming a positive feedback loop^81^. Integrin αvβ5 was reported to activate ERK signaling and to subsequently enhance the dedifferentiation of in vitro cultured primary human articular chondrocytes^82^. In addition, the Notch pathway is involved in the regulation of matrix metalloproteinase 13 (MMP-13) activity and the differentiation of human articular chondrocytes in vitro^83^. In rabbit articular chondrocytes in vitro, 2-deoxy-D-glucose (2DG) induces dedifferentiation via the β-catenin pathway^84^. A recent study revealed a feed-forward loop driven by USP14 (a deubiquitinating enzyme) and the NF-kappa B pathway that enhances the effect of IL-1β on the dedifferentiation of human chondrocytes in vitro^85^.

The architecture of the actin cytoskeleton modulates the phenotype of chondrocytes during dedifferentiation^86^. The disruption of the actin cytoskeleton inhibits the dedifferentiation of in vitro cultured rabbit chondrocytes by blocking PKCα and –ζ signaling^87^. The integrity of the actin cytoskeleton is related to the differentiated phenotype of chondrocytes through the PI3K/Akt and p38 pathways^88^. In addition, the Rho family GTPase ROCK plays a key role in organizing the actin cytoskeleton^89^. Treatment with a ROCK inhibitor alters human chondrocyte morphology in vitro and activates SOX9 and chondrogenic gene expression, suggesting the suppression of dedifferentiation^90^. Berberine, a compound widely present in many plants, reorganizes the actin cytoskeleton and subsequently induces dedifferentiation via PI3K/Akt and p38 pathways^91^. Focal adhesion kinase (FAK) is involved in cell adhesion, migration and cell proliferation. The role of FAK in the chondrocytic dedifferentiation process was investigated by Shin et al.^92^ An increasing number of passages in monolayer culture resulted in higher levels of focal adhesion complexes. In addition, FAK knockdown facilitates the restoration of the expression of cartilage-specific genes, including Col II, aggrecan and SOX9, in rat dedifferentiated chondrocytes in vitro. In another study, FAK knockdown inhibited porcine chondrocyte proliferation in vitro, and FAK was required to modulate the expression of Col II^93^. Ding and colleagues^94^ further explored the effects of spreading areas and aspect ratios of single chondrocytes undergoing dedifferentiation using a micropatterning technique in vitro. Rat chondrocytes incubated under conditions of larger sizes and higher aspect ratios preferentially undergo dedifferentiation.

### Implications from recent studies of dedifferentiation

The information above provides interesting implications. First, dedifferentiation may have either positive or negative consequences to tissue repair in different tissue/organs. For example, after injury, dedifferentiated cardiomyocytes were preferentially protected from apoptosis; they can survive and re-enter cell cycle for tissue repair^16^. Meanwhile, dedifferentiated chondrocytes gradually lose the ability of proliferation and undergo hypertrophy and eventually apoptosis^95^. Therefore, understanding the impact of cell dedifferentiation in specific tissue is essential for devising further therapeutic strategies—such as induction versus suppression (and to what extent). To date, much remains unknown about the causes and consequences of dedifferentiation under specific physiological or pathological circumstances, which requires more mechanistic studies at different levels.

Second, it should be noted that the findings from non-human models provide valuable evidence for assessing the application potential of dedifferentiation for future trials and translations in humans. Many studies show that a main reason for the huge difference of regenerative capacity between amphibians and mammals is the induction of dedifferentiation, which does not often occur after injury in mammals^8^. Inspired by the studies on dedifferentiation of amphibians such as newt, researchers found that murine myotubes could dedifferentiate to a certain extent when stimulated by an extract obtained from newt regenerating limbs^96^. Also, partial dedifferentiation of Schwann cells can be induced by local factors such as hormones and neurotrophic factors^97^. These results suggest that such extrinsic signals can play an important role in dedifferentiation. Although current interpretations remain on a rather speculative level and await further verification, in vitro investigations on dedifferentiation provide valuable insights into the molecular mechanisms and the pathways associated with cell dedifferentiation, with extensive in vivo studies in demand.

Third, by exploring the regenerative phenomena in invertebrates and amphibians, researchers have uncovered the cellular and molecular mechanisms to develop regenerative strategies for humans. Dedifferentiation and trans-differentiation, part of the physiological response to injury in numerous organs, are mechanistically associated with natural regeneration^98^. Dedifferentiation refers to a reversion of a fully differentiated cell within its own lineage, which allows the cell to proliferate again to replace those damaged cells in many cases^99^. Trans-differentiation, first reported in the newt for regeneration of lens over 100 years ago, involves dedifferentiation in the first step and subsequent differentiation into another cell type. It may also be observed during experimental induction of trans-differentiation that cells can directly trans-differentiate to a distinct lineage through an “unnatural” intermediate phase. Thus, a strategy for regenerative medicine would be either (i) to induce dedifferentiation and then proliferation of target cells or (ii) to stimulate trans-differentiation of cells into the desired cell types. In either case, a clear dissection of the changes and impacts of the genes involved in dedifferentiation is fundamental.

### Engineering approaches to regulate dedifferentiation for therapeutic purposes

Although mesenchymal stem cells (MSCs) are promising “seed” cells in tissue regeneration owing to their excellent characteristics such as self-renewal capacity^100^, immunoregulatory function^101^, and multilineage differentiation potential^102^, MSC-based therapies for tissue regeneration have not yet been accepted clinically^103^. Current efforts focus on the development of bioreactor system, which could combine scaffolds, biochemical factors, and mechanical stimuli to simulate the in vivo microenvironment so that MSCs-based cellular products can meet the requirements for clinical use^104^. Recent studies have provided evidence that dedifferentiation is a promising approach for tissue regeneration^4^. An increasing number of trials have used engineering approaches to regulate dedifferentiation.

#### Creation of tissue microenvironment

Scaffold materials, which can provide a three-dimensional culture condition that more closely resembles the in vivo environment, play a critical role in the biological characteristics and proliferation rate of seeding cells, subsequently determining the fate and function of cells^105^. Recently, researchers have focused on designing and preparing innovative biomaterials to induce dedifferentiation. Yahalom-Ronen et al.^15^ established an in vitro culture system for murine P1 neonatal cardiomyocytes. They constructed softer substrates to stimulate dedifferentiation and the cell cycle re-entry of cardiomyocytes, suggesting that the development of soft scaffolds might facilitate the regeneration of the adult human heart. A keratin hydrogel was created and used to repair a 1-cm defect in rat sciatic nerve injury models^106^. Compared with Matrigel, the keratin hydrogel group induced more rapid dedifferentiation of Schwann cells, leading to the faster activation of macrophages and more efficient clearance of myelin debris and ECM remodeling. Thus, the positive regulation of Schwann cell dedifferentiation encourages effective nerve regeneration.

**Fig. 3:**
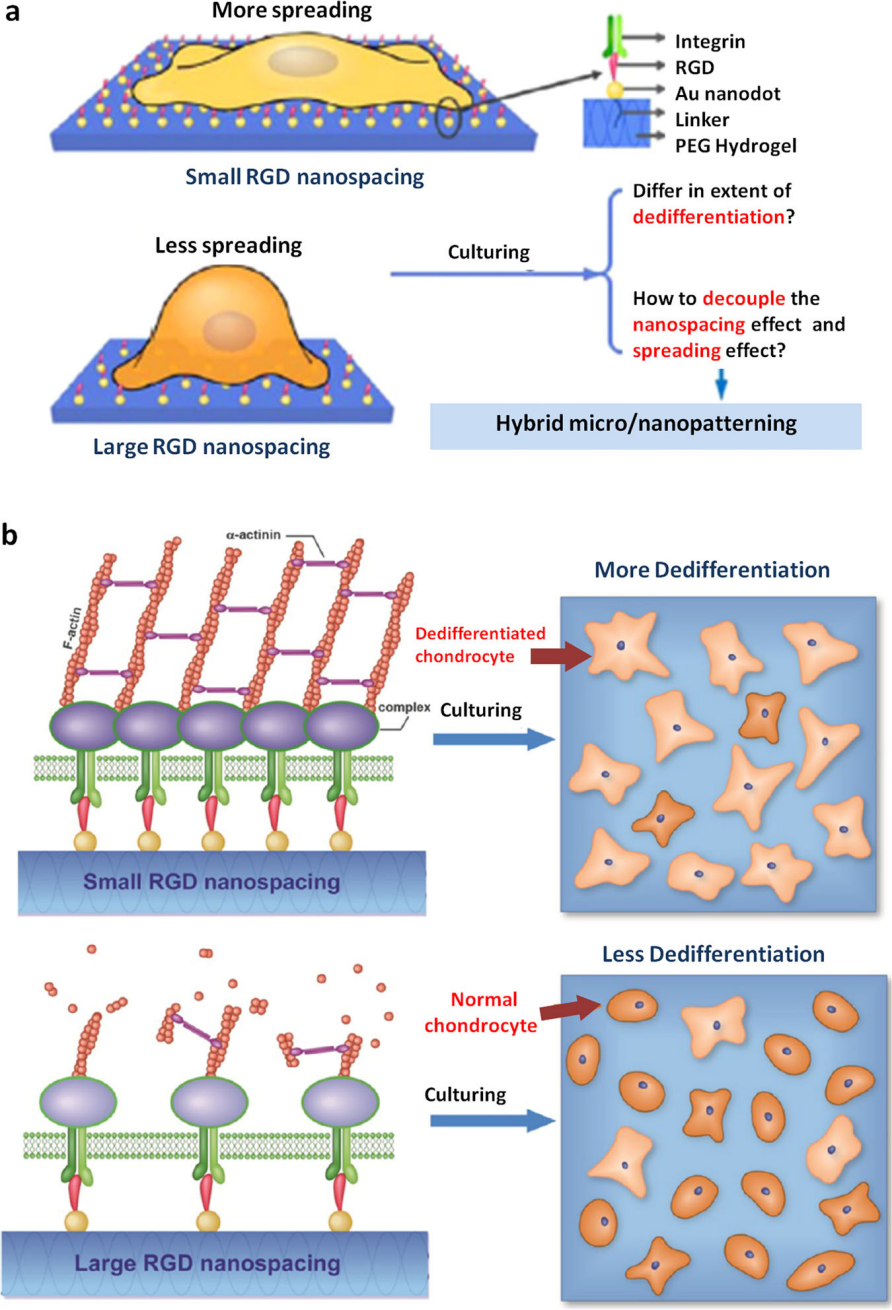
Effects of a nanoscale spatial arrangement of RGD on dedifferentiation of chondrocytes. **a** Schematic illustrating the experimental design. PEG hydrogel surfaces were nanopatterned with RGD peptides and then cultured with chondrocytes to explore the effect of material techniques of nanopatterning on the dedifferentiation of chondrocytes. **b** Schematic illustrating the effects of RGD nanopatterns on the dedifferentiation of chondrocytes. RGD with a nanospacing greater than 70 nm is beneficial for maintaining the chondrocyte phenotype. Reprinted (adapted) with permission from Li et al.^114^, copyright 2015, American Chemistry Society.

On the other hand, many studies of biomaterials have focused on preventing the dedifferentiation of chondrocytes, as the dedifferentiation of chondrocytes severely compromises the therapeutic outcome of cartilage repair. Dedifferentiation is induced when chondrocytes are exposed to degradative enzymes during passaging or incubated in a standard culture flask with a flat and rigid culture surface. Through a series of studies, Quinn and colleagues^107^ developed a “continuous expansion” (CE) in vitro culture technique that avoids repeated passaging and maintains the chondrocytic phenotype. Using this new method, bovine chondrocytes were cultured on high-extension silicone rubber (HESR) dishes with continuously expanding and elastic properties. Furthermore, the authors functionalized the HESR dishes with a cartilage ECM extract from a cow^108^. HESR dishes functionalized with a cartilage extract were more effective at inhibiting chondrocytic dedifferentiation than control dishes. Another group further developed biomimetic materials using decellularized ECM derived from human articular chondrocytes^14^. These biomaterials were more effective at maintaining the phenotype and enhancing the proliferation of chondrocytes than standard culture plates. Moreover, similar results were obtained using MSC-derived ECM^109,110^, indicating that a cell type-specific ECM is more similar to the native microenvironment and reduces the dedifferentiation of chondrocytes. Gelatin methacryloyl/hyaluronic acid methacrylate hydrogels displayed their positive effects on down-regulation of dedifferentiation gene markers^111^. In addition, a biomimetic hydrogel was prepared based on alginate and tuned by altering the concentration of chlorosulfonic acid^112^. The sulfation of alginate, which mimics proteoglycans in cartilage, promotes in vitro bovine chondrocyte proliferation, increase cartilage matrix deposition, and inhibits the expression of dedifferentiation markers. A blended gel composed of alginate and collagen also provided a preferable gel matrix environment for the suppression of rat chondrocyte dedifferentiation in vitro^113^. More interestingly, Ding and colleagues^114^ concentrated on the effects of external nanoscale features on the dedifferentiation of in vitro cultured primary rat chondrocytes. Poly(ethylene glycol) (PEG) hydrogels were modified with nanopatterned arginine–glycine–aspartate (RGD) and used to culture chondrocytes (Fig. 3). The nanoscale distribution of RGD revealed a crucial and independent role for this material in regulating cell dedifferentiation. Overall, all of these aspects should be specifically considered when designing and optimizing advanced biomaterials for regenerative medicine.

#### Biochemical regulation

Numerous biomolecules have shown the potential in modulating dedifferentiation and have been applied in regenerative medicine. The methods used to deliver biomolecules mainly include—(i) direct addition in the form of recombinant proteins and (ii) gene delivery of their complementary DNAs (cDNAs) using viral or nonviral vectors.

MMPs, which are capable of degrading ECM proteins, regulate Schwann cell signaling and mitosis. As shown in a study by the Shubayev group, MMP-9 inhibits Schwann cell mitosis during nerve regeneration, while the MMP inhibitor, GM6001, counteracts the effect of MMP-9 and further increases the division of murine Schwann cells^115^. Based on these findings, the group subsequently performed in vivo experiments; GM6001 was injected into rats immediately after sciatic nerve crush to stimulate Schwann cell division, reduce myelin protein expression and subsequently support axonal regeneration^116^. In another interesting study, leukemia inhibitory factor (LIF), a promyelination factor, was encapsulated in nanoparticle carriers and delivered to enhance remyelination and promote nerve regeneration^117^, highlighting the feasibility of biochemically regulating tissue development with engineering approaches. Similarly, recombinant human midkine was added to the growth medium of rat chondrocytes for in vitro monolayer expansion and not only promoted cell proliferation but also suppressed dedifferentiation, avoiding a shift in the chondrocyte to fibroblast phenotype and decreasing the expression of chondrocytic genes^118^. In addition, andrographolide, the main active component of the traditional Chinese medicine *Andrographis paniculata*, also prevents the dedifferentiation of rabbit articular chondrocytes in vitro, and 3 μM was reported to be the optimal dose^119^. Although positive effects have been observed by directly supplementing culture media with biomolecules, this method will result in instability in vivo, limiting further clinical applications. Therefore, gene-transfer strategies appear to be a promising alternative.

Two main types of gene delivery vectors have bee identified: viral vectors, such as adenoviral vectors and lentiviral vectors, and nonviral vectors, such as polymers and liposomes. Viral vectors have better transfection efficiencies while nonviral vectors have advantages, such as ease of synthesis and low immunogenicity. Endothelial nitric oxide (eNO) plays a pivotal role in regulating Schwann cell dedifferentiation. NO synthesized by eNO synthase decelerates axon regeneration after crush of the XII nerve^120^. Therefore, researchers have constructed adenoviral vectors to express a truncated mutant form of eNO synthase followed by an intraneural injection of the adenovirus^121^. Rat Schwann cell dedifferentiation was induced, as evidenced by the overexpression of dedifferentiated Schwann cell marker growth-associated protein 43 and an increase in the bands of Bungner, the cellular substrate for guiding growing axons. On the other hand, various biomolecules are being developed to inhibit the dedifferentiation of chondrocytes. Based on the results of the previous studies showing that IL-1β induces the dedifferentiation of chondrocytes and is inhibited by cytokine response modifier A (CrmA)^81,122,123^, hyaluronic acid-chitosan microspheres were designed for the controlled release of CrmA^124^. Rat chondrocytes treated with these microspheres in vitro maintain the chondrocytic phenotype and production of cartilage-specific proteins even after cocultured with IL-1β. Additionally, chondrocytes expressing wild-type p53-inducible phosphatase (Wip1) following microporation generally retain a chondrocyte phenotype for a longer time during in vitro expansion^125^.

A “Tet-Off” transgenic mouse model was established by Tzahor and colleagues^126^ to modulate the expression of a constitutively active (ca) form of ErbB2 in murine cardiomyocytes following the administration of the tetracycline analog doxycycline (DOX). Notably, caErbB2 promotes the dedifferentiation and subsequently induces cell division, highlighting the proliferative and regenerative potential of adult cardiomyocytes.

#### Molecular/cellular modulation

**Fig. 4:**
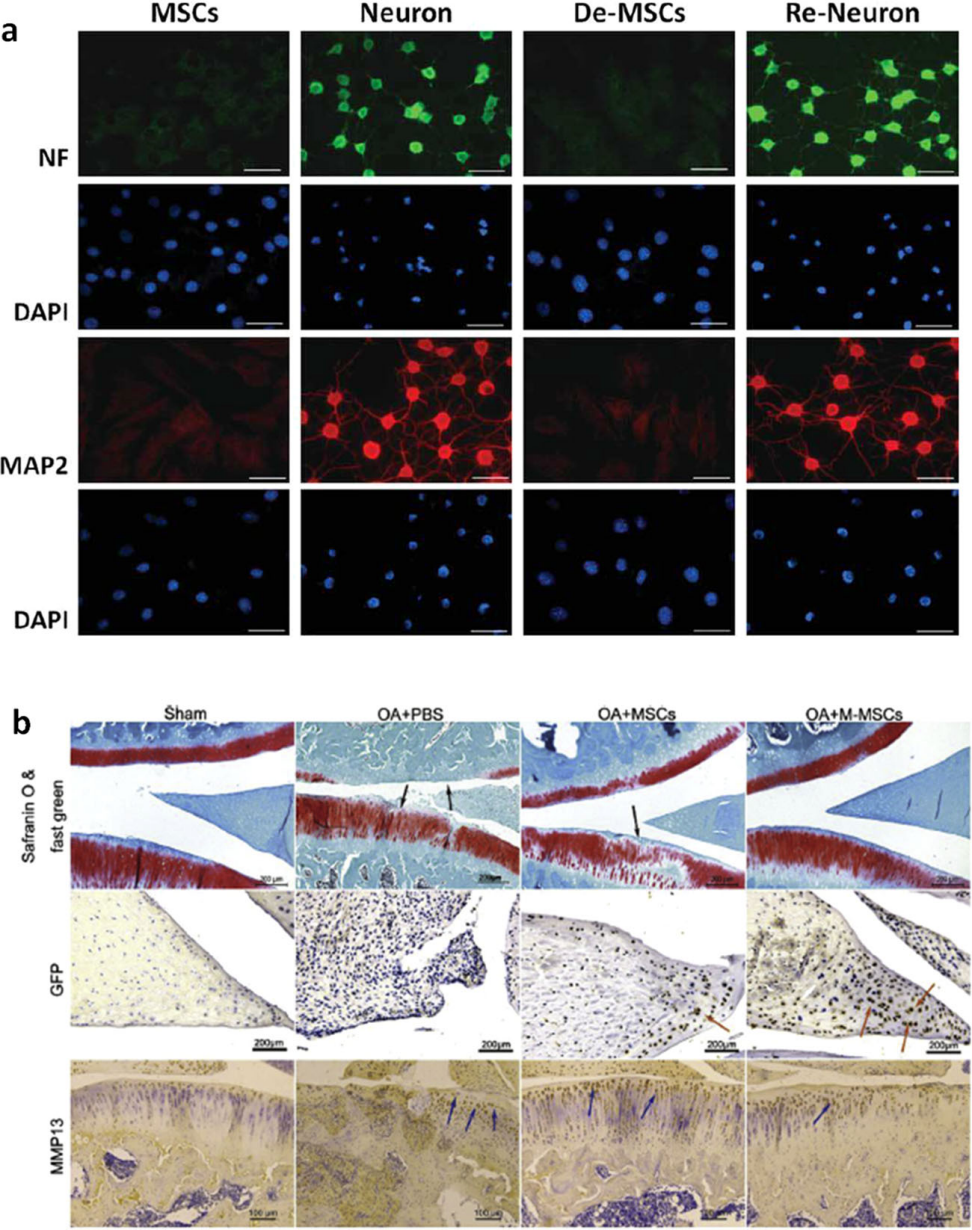
Dedifferentiation-reprogrammed mesenchymal stem cells with promising therapeutic potential for cartilage and nerves. **a** Immunofluorescence staining revealed the preferential neuronal differentiation of De-MSCs, as evidenced by the higher expression of neuronal markers, including neurofilament-M (NF-M) and microtubule-associated protein 2 (MAP2) (used with permission from John Wiley and Sons/Hsiao et al.^127^). **b** Histology and immunohistochemistry revealed that the M-MSC group exhibited a greater therapeutic effect on OA, as evidenced by the increased ECM deposition, increased number of GFP-positive cells, and reduction in MMP-13 expression^129^ (Copyright 2017, with permission from Elsevier).

Studies have shown that MSC-derived neurons dedifferentiate into MSCs after the removal of extrinsic stimuli. Liu et al.^127^ explored the therapeutic potential of the dedifferentiated MSCs (De-MSCs) in neuron repair (Fig. 4a). In vitro monoclonal rat MSCs were harvested and induced to differentiate into neurons with modified neuronal medium for 24–48 h. Then, the De-MSCs were obtained after incubation in complete MSC medium for another 48 h. The researchers were the first to show that De-MSCs tended to undergo neuronal differentiation, as evidenced by genetic and functional assays, compared to uncommitted MSCs. Moreover, in vitro and in vivo, De-MSCs show increased cell survival, greater resistance to a hostile environment and a higher efficacy of neuronal differentiation, suggesting a better therapeutic effect. In addition to being derived from the same lineage, dedifferentiation appeared to be a prerequisite for bone marrow-derived neurons to differentiate into a different lineage^128^. Moreover, direct translineage differentiation from neuronal cells to epithelial was not observed under appropriate defined conditions. Interestingly, the dedifferentiation-reprogramming method was also effective in chondrogenesis. According to Li et al.^129^, rat MSCs in vitro that are incubated in chondrogenic induction medium undergo chondrogenesis and then are allowed to grow and subculture in growth medium to expand and dedifferentiate (Fig. 4b). Based on these findings, chondrogenically manipulated MSCs (M-MSCs) harvested in this manner display higher viability and chondrogenic potential.

Other factors exerting an inhibitory effect on dedifferentiation are also studied. A study by Li et al. reveals that DNA methylation is an important regulator of chondrocyte dedifferentiation. The authors applied the DNA methylation inhibitor 5-azacytidine (5-AzaC) to human chondrocytes in vitro, resulting in an increase in the DNA methylation level of the Col I-A1 promoter, which slowed chondrocyte dedifferentiation^130^. Low-dose γ-radiation also effectively inhibits the IL-1β-induced dedifferentiation of in vitro cultured primary rabbit articular chondrocytes^131^. Other researchers have also delivered biomolecules^132,133^ or optimized culture conditions, such as materials^134,135^, oxygen tension^136,137^, and mechanical stimulation^138^, to drive efficient redifferentiation of dedifferentiated chondrocytes for use in regenerative medicine.

## Conclusions and future perspectives

Discovering the interesting phenomenon of cell dedifferentiation, followed by uncovering its underlying mechanisms, opens new avenues for controlling cell fate for regenerative applications. It is possible that in different tissue/organs, dedifferentiation occurs in various frequencies and to different extents; and perhaps much remains unknown about the causes and consequences of this phenomenon under specific physiological or pathological circumstances. However, there must be a reason that the organisms develop the capability of dedifferentiation through the long period of evolution—to adapt, to survive, to heal, to evade from dangers, and to evolve. As such, recapitulating dedifferentiation in regenerative medicine may provide a new, rational, evolution-inspired strategy to bypass inherent limitations with therapeutic cells (such as inability to proliferate) and initiate tissue development under better control.

Future studies can be directed in three aspects. First, more mechanistic studies at different levels are required to understand the initiation of cell dedifferentiation. Genetic, epigenetic, transcriptional, metabolic, and mechanobiological controls of the cell fate decision towards dedifferentiation are all worth investigation, which will offer comprehensive clues for designing engineering tools. Second, tissue regeneration involves multiple cell types of different lineages and characteristics, especially in the case of a complex organ such as the liver, kidney, and heart. It is very likely that the change in the behavior of one cell type affects the function of another. Hence, the consequence of engineered dedifferentiation of a group of target cells on other cells in the tissue niche should be carefully examined. Any potential adverse effects—either short-term (e.g., cell death, acute inflammation, and off-target effect) or long-term (e.g., carcinogenicity caused by uncontrolled cell proliferation after induced dedifferentiation) risks—need to be avoided. Third, for any practice in which cell dedifferentiation demonstrates preliminary signs of safety and effectiveness, extensive work is demanded to standardize the protocol. Major questions include: how far should the cells be reversed along the way of dedifferentiation, how many percentages of the cells are effectively induced to dedifferentiate, and when is the best timing to apply the dedifferentiated cells into tissue repair. As such, exciting future work lies ahead from biological, clinical, and engineering perspectives for translating the discovery of cell dedifferentiation into tissue regeneration.

## References

[1] Jopling C (2010). Zebrafish heart regeneration occurs by cardiomyocyte dedifferentiation and proliferation. Nature.

[2] Zhang L (2016). Fifteen new earthworm mitogenomes shed new light on phylogeny within the Pheretima complex. Sci. Rep..

[3] Sarig R, Tzahor E (2017). The cancer paradigms of mammalian regeneration: can mammals regenerate as amphibians?. Carcinogenesis.

[4] Jopling C, Boue S, Izpisua Belmonte JC (2011). Dedifferentiation, transdifferentiation and reprogramming: three routes to regeneration. Nat. Rev. Mol. Cell Biol..

[5] Spence JR (2011). Directed differentiation of human pluripotent stem cells into intestinal tissue in vitro. Nature.

[6] Charlier E (2019). Chondrocyte dedifferentiation and osteoarthritis (OA). Biochem. Pharmacol..

[7] Bianchi VJ (2019). Redifferentiated chondrocytes in fibrin gel for the repair of articular cartilage lesions. Am. J. Sports Med..

[8] Pesaresi M, Sebastian-Perez R, Cosma MP (2018). Dedifferentiation, transdifferentiation and cell fusion: invivo reprogramming strategies for regenerative medicine. FEBS J..

[9] Zhang CP (2012). Wnt/ss-catenin signaling is critical for dedifferentiation of aged epidermal cells in vivo and in vitro. Aging Cell.

[10] Laadhar L (2014). Wnt signaling is involved in human articular chondrocyte dedifferentiation in vitro. Osteoporos. Int..

[11] Kohler EE (2014). Low-dose 6-bromoindirubin-3‘-oxime induces partial dedifferentiation of endothelial cells to promote increased neovascularization. Stem Cells.

[12] Fan Y (2017). Macrophage migration inhibitory factor triggers vascular smoothmuscle cell dedifferentiation by a p68-serum response factor axis. Cardiovascular Res..

[13] Eo SH, Kim DW, Choi SY, Kim HA, Kim SJ (2015). PEP-1-SIRT2 causes dedifferentiation and COX-2 expression via the MAPK pathways in rabbit articular chondrocytes. Exp. Cell Res..

[14] Mao Y (2019). Extracellular matrix derived from chondrocytes promotes rapid expansion of human primary chondrocytes in vitro with reduced dedifferentiation. Acta Biomater..

[15] Yahalom-Ronen, Y., Rajchman, D., Sarig, R., Geiger, B. & Tzahor, E. Reduced matrix rigidity promotes neonatal cardiomyocyte dedifferentiation, proliferation and clonal expansion. *Elife* **4**, e07455 (2015). 10.7554/eLife.07455 PMC4558647 26267307

[16] Szibor M, Poling J, Warnecke H, Kubin T, Braun T (2014). Remodeling and dedifferentiation of adult cardiomyocytes during disease and regeneration. Cell. Mol. Life Sci..

[17] Eguizabal C, Montserrat N, Veiga A, Belmonte JCI (2013). Dedifferentiation, transdifferentiation, and reprogramming: future directions in regenerative medicine. Semin. Reprod. Med..

[18] Porrello ER (2013). Regulation of neonatal and adult mammalian heart regeneration by the miR-15 family. Proc Natl Acad. Sci. USA.

[19] Ali H, Braga L, Giacca M (2020). Cardiac regeneration and remodelling of the cardiomyocyte cytoarchitecture. FEBS J..

[20] Wang CY (2019). Cardiomyocyte dedifferentiation and remodeling in 3D scaffolds to generate the cellular diversity of engineering cardiac tissues. Biomater. Sci..

[21] Taegtmeyer H, Sen S, Vela D (2010). Return to the fetal gene program: a suggested metabolic link to gene expression in the heart. Ann. N. Y. Acad. Sci..

[22] Zhang, Y. Q. et al. Single-cell imaging and transcriptomic analyses of endogenous cardiomyocyte dedifferentiation and cycling. *Cell Discov.* **5**, 30 (2019). 10.1038/s41421-019-0095-9 PMC6547664 31231540

[23] Poling J (2012). The Janus face of OSM-mediated cardiomyocyte dedifferentiation during cardiac repair and disease. Cell Cycle.

[24] Dispersyn GD, Geuens E, Ver Donck L, Ramaekers FC, Borgers M (2001). Adult rabbit cardiomyocytes undergo hibernation-like dedifferentiation when co-cultured with cardiac fibroblasts. Cardiovasc Res..

[25] Kubin T (2011). Oncostatin M is a major mediator of cardiomyocyte dedifferentiation and remodeling. Cell Stem Cell.

[26] Ikeda S (2019). Hippo deficiency leads to cardiac dysfunction accompanied by cardiomyocyte dedifferentiation during pressure overload. Circulation Res..

[27] Ruoslahti E (1997). Stretching is good for a cell. Science.

[28] Tanaka EM, Gann AA, Gates PB, Brockes JP (1997). Newt myotubes reenter the cell cycle by phosphorylation of the retinoblastoma protein. J. Cell Biol..

[29] Wohlschlaeger J (2010). Reversible regulation of the, retinoblastoma protein/E2F-1 pathway during “reverse cardiac remodelling” after ventricular unloading. J. Heart Lung Transplant..

[30] MacLellan WR (2005). Overlapping roles of pocket proteins in the myocardium are unmasked by germ line deletion of p130 plus heart-specific deletion of Rb. Mol. Cell Biol..

[31] Zaglia T (2009). Cardiac interstitial cells express GATA4 and control dedifferentiation and cell cycle re-entry of adult cardiomyocytes. J. Mol. Cell Cardiol..

[32] Zhang GY (2019). GRP78 (glucose-regulated protein of 78 kDa) promotes cardiomyocyte growth through activation of GATA4 (GATA-binding protein 4). Hypertension.

[33] Carr MJ, Johnston APW (2017). Schwann cells as drivers of tissue repair and regeneration. Curr. Opin. Neurobiol..

[34] Webber C, Zochodne D (2010). The nerve regenerative microenvironment: early behavior and partnership of axons and Schwann cells. Exp. Neurol..

[35] Warner LE (1998). Mutations in the early growth response 2 (EGR2) gene are associated with hereditary myelinopathies. Nat. Genet..

[36] Jessen KR, Mirsky R (2008). Negative regulation of myelination: relevance for development, injury, and demyelinating disease. Glia.

[37] Jessen KR, Mirsky R (2005). The origin and development of glial cells in peripheral nerves. Nat. Rev. Neurosci..

[38] Woodhoo A (2009). Notch controls embryonic Schwann cell differentiation, postnatal myelination and adult plasticity. Nat. Neurosci..

[39] Napoli I (2012). A central role for the ERK-signaling pathway in controlling Schwann cell plasticity and peripheral nerve regeneration in vivo. Neuron.

[40] Groh J (2015). CSF-1-activated macrophages are target-directed and essential mediators of Schwann cell dedifferentiation and dysfunction in Cx32-deficient mice. Glia.

[41] Mirsky R (2008). Novel signals controlling embryonic Schwann cell development, myelination and dedifferentiation. J. Peripheral Nerv. Syst.: JPNS.

[42] Parkinson DB (2008). c-Jun is a negative regulator of myelination. J. Cell Biol..

[43] Norrmen C (2018). mIORC1 is transiently reactivated in injured nerves to promote c-Jun elevation and Schwann cell dedifferentiation. J. Neurosci..

[44] Fontana X (2012). c-Jun in Schwann cells promotes axonal regeneration and motoneuron survival via paracrine signaling. J. Cell Biol..

[45] Arthur-Farraj PJ (2012). c-Jun reprograms Schwann cells of injured nerves to generate a repair cell essential for regeneration. Neuron.

[46] Yang DP (2012). p38 MAPK activation promotes denervated Schwann cell phenotype and functions as a negative regulator of Schwann cell differentiation and myelination. J. Neurosci..

[47] Jung J (2011). Actin polymerization is essential for myelin sheath fragmentation during Wallerian degeneration. J. Neurosci..

[48] Shin YK (2013). The Neuregulin-Rac-MKK7 pathway regulates antagonistic c-jun/Krox20 expression in Schwann cell dedifferentiation. Glia.

[49] Shin YH, Lee SJ, Jung J (2013). Extracellular ATP inhibits Schwann cell dedifferentiation and proliferation in an ex vivo model of Wallerian degeneration. Biochem. Biophys. Res. Commun..

[50] Monje PV, Soto J, Bacallao K, Wood PM (2010). Schwann cell dedifferentiation is independent of mitogenic signaling and uncoupled to proliferation: role of cAMP and JNK in the maintenance of the differentiated state. J. Biol. Chem..

[51] Viader A, Chang LW, Fahrner T, Nagarajan R, Milbrandt J (2011). MicroRNAs modulate Schwann cell response to nerve injury by reinforcing transcriptional silencing of dedifferentiation-related genes. J. Neurosci..

[52] Corti S (2012). Direct reprogramming of human astrocytes into neural stem cells and neurons. Exp. Cell Res..

[53] Moon JH (2011). Nanog-induced dedifferentiation of p53-deficient mouse astrocytes into brain cancer stem-like cells. Biochem. Biophys. Res. Commun..

[54] Yang H, Cheng XP, Li JW, Yao Q, Ju G (2009). De-differentiation response of cultured astrocytes to injury induced by scratch or conditioned culture medium of scratch-insulted astrocytes. Cell Mol. Neurobiol..

[55] Yang H (2010). Evidence for heterogeneity of astrocyte de-differentiation in vitro: astrocytes transform into intermediate precursor cells following induction of ACM from scratch-insulted astrocytes. Cell Mol. Neurobiol..

[56] Sher F, Boddeke E, Copray S (2011). Ezh2 expression in astrocytes induces their dedifferentiation toward neural stem cells. Cell Reprogram.

[57] Yang H (2011). ErbB2 activation contributes to de-differentiation of astrocytes into radial glial cells following induction of scratch-insulted astrocyte conditioned medium. Neurochem. Int..

[58] Kosaka N (2006). FGF-4 regulates neural progenitor cell proliferation and neuronal differentiation. FASEB J..

[59] Feng GD (2014). Fibroblast growth factor 4 is required but not sufficient for the astrocyte dedifferentiation. Mol. Neurobiol..

[60] Yang H (2012). Sonic hedgehog released from scratch-injured astrocytes is a key signal necessary but not sufficient for the astrocyte de-differentiation. Stem Cell Res..

[61] Sirko S (2013). Reactive glia in the injured brain acquire stem cell properties in response to sonic hedgehog. [corrected]. Cell Stem Cell.

[62] Hill, S. A. et al. Sonic hedgehog signaling in astrocytes mediates cell type-specific synaptic organization. *Elife* **8**, e45545 (2019). 10.7554/eLife.45545 PMC6629371 31194676

[63] Sanchez MA, Sullivan GM, Armstrong RC (2018). Genetic detection of Sonic hedgehog (Shh) expression and cellular response in the progression of acute through chronic demyelination and L updates amok for remyelination. Neurobiol. Dis..

[64] Ugbode CI, Smith I, Whalley BJ, Hirst WD, Rattray M (2017). Sonic hedgehog signalling mediates astrocyte crosstalk with neurons to confer neuroprotection. J. Neurochemis..

[65] Yang H (2019). Sonic hedgehog effectively improves Oct4-mediated reprogramming of astrocytes into neural stem cells. Mol. Ther..

[66] Hall, A. C. The role of chondrocyte morphology and volume in controlling phenotypeimplications for osteoarthritis, cartilage repair, and cartilage engineering. *Curr. Rheumatol. Rep.* **21**, 38 (2019). 10.1007/s11926-019-0837-6 PMC6571082 31203465

[67] Wu L (2014). Extracellular matrix domain formation as an indicator of chondrocyte dedifferentiation and hypertrophy. Tissue Eng. Part C. Methods.

[68] Diaz-Romero J, Nesic D, Grogan SP, Heini P, Mainil-Varlet P (2008). Immunophenotypic changes of human articular chondrocytes during monolayer culture reflect bona fide dedifferentiation rather than amplification of progenitor cells. J. Cell Physiol..

[69] Kruger M, Kruger JP, Kinne RW, Kaps C, Endres M (2015). Are surface antigens suited to verify the redifferentiation potential and culture purity of human chondrocytes in cell-based implants. Tissue Cell.

[70] Hong E, Reddi AH (2013). Dedifferentiation and redifferentiation of articular chondrocytes from surface and middle zones: changes in microRNAs-221/-222, -140, and -143/145 expression. Tissue Eng. Part A.

[71] Sliogeryte K, Botto L, Lee DA, Knight MM (2016). Chondrocyte dedifferentiation increases cell stiffness by strengthening membrane-actin adhesion. Osteoarthr. Cartil..

[72] Minegishi Y, Hosokawa K, Tsumaki N (2013). Time-lapse observation of the dedifferentiation process in mouse chondrocytes using chondrocyte-specific reporters. Osteoarthr. Cartil..

[73] Rosenzweig DH, Ou SJ, Quinn TM (2013). P38 mitogen-activated protein kinase promotes dedifferentiation of primary articular chondrocytes in monolayer culture. J. Cell Mol. Med..

[74] Yu SM, Yeo HJ, Choi SY, Kim SJ (2016). Cytokine-induced apoptosis inhibitor-1 causes dedifferentiation of rabbit articular chondrocytes via the ERK-1/2 and p38 kinase pathways. Int. J. Biochem. Cell Biol..

[75] Yu SM, Choi YJ, Kim SJ (2018). PEP-1-glutaredoxin-1 induces dedifferentiation of rabbit articular chondrocytes by the endoplasmic reticulum stress-dependent ERK-1/2 pathway and the endoplasmic reticulum stress-independent p38 kinase and PI-3 kinase pathways. Int. J. Biol. Macromolecules.

[76] Han Y, Kim SJ (2016). Simvastatin induces differentiation of rabbit articular chondrocytes via the ERK-1/2 and p38 kinase pathways. Exp. Cell Res..

[77] Yu SM, Kim SJ (2015). The thymoquinone-induced production of reactive oxygen species promotes dedifferentiation through the ERK pathway and inflammation through the p38 and PI3K pathways in rabbit articular chondrocytes. Int. J. Mol. Med..

[78] Lee WK, Yu SM, Cheong SW, Sonn JK, Kim SJ (2008). Ectopic expression of cyclooxygenase-2-induced dedifferentiation in articular chondrocytes. Exp. Mol. Med..

[79] Yu SM, Kim SJ (2015). Salinomycin causes dedifferentiation via the extracellular signal-regulated kinase (ERK) pathway in rabbit articular chondrocytes. J. Pharm. Sci..

[80] Martinon F, Tschopp J (2004). Inflammatory caspases: linking an intracellular innate immune system to autoinflammatory diseases. Cell.

[81] Hong EH (2011). Nicotinamide phosphoribosyltransferase is essential for interleukin-1beta-mediated dedifferentiation of articular chondrocytes via SIRT1 and extracellular signal-regulated kinase (ERK) complex signaling. J. Biol. Chem..

[82] Fukui N (2011). alphavbeta5 integrin promotes dedifferentiation of monolayer-cultured articular chondrocytes. Arthritis Rheum..

[83] Sassi N (2014). Notch signaling is involved in human articular chondrocytes de-differentiation during osteoarthritis. J. Receptors Signal. Transduct..

[84] Yu SM, Kim HA, Kim SJ (2010). 2-Deoxy-D-glucose regulates dedifferentiation through beta-catenin pathway in rabbit articular chondrocytes. Exp. Mol. Med..

[85] Li MG, Zhao JQ, Jia LG (2019). USP14-mediated I kappa B alpha degradation exacerbates NF-kappa B activation and IL-1 beta-stimulated chondrocyte dedifferentiation. Life Sci..

[86] Parreno J (2017). Interplay between cytoskeletal polymerization and the chondrogenic phenotype in chondrocytes passaged in monolayer culture. J. Anat..

[87] Kim SJ, Hwang SG, Kim IC, Chun JS (2003). Actin cytoskeletal architecture regulates nitric oxide-induced apoptosis, dedifferentiation, and cyclooxygenase-2 expression in articular chondrocytes via mitogen-activated protein kinase and protein kinase C pathways. J. Biol. Chem..

[88] Park EH (2008). Integrity of the cortical actin ring is required for activation of the PI3K/Akt and p38 MAPK signaling pathways in redifferentiation of chondrocytes on chitosan. Cell Biol. Int..

[89] Burridge K, Wennerberg K (2004). Rho and Rac take center stage. Cell.

[90] Matsumoto E, Furumatsu T, Kanazawa T, Tamura M, Ozaki T (2012). ROCK inhibitor prevents the dedifferentiation of human articular chondrocytes. Biochem. Biophys. Res. Commun..

[91] Yu SM (2016). Berberine induces dedifferentiation by actin cytoskeleton reorganization via phosphoinositide 3-kinase/Akt and p38 kinase pathways in rabbit articular chondrocytes. Exp. Biol. Med. (Maywood).

[92] Shin H (2016). Focal adhesion assembly induces phenotypic changes and dedifferentiation in chondrocytes. J. Cell Physiol..

[93] Kim YH, Lee JW (2009). Targeting of focal adhesion kinase by small interfering RNAs reduces chondrocyte redifferentiation capacity in alginate beads culture with type II collagen. J. Cell Physiol..

[94] Cao B, Peng R, Li Z, Ding J (2014). Effects of spreading areas and aspect ratios of single cells on dedifferentiation of chondrocytes. Biomaterials.

[95] Hwang HS, Kim HA (2015). Chondrocyte apoptosis in the pathogenesis of osteoarthritis. Int. J. Mol. Sci..

[96] McGann CJ, Odelberg SJ, Keating MT (2001). Mammalian myotube dedifferentiation induced by newt regeneration extract. Proc. Natl Acad. Sci. USA.

[97] Chen ZL, Yu WM, Strickland S (2007). Peripheral regeneration. Annu. Rev. Neurosci..

[98] Merrell AJ, Stanger B (2016). Adult cell plasticity in vivo: de-differentiation and transdifferentiation are back in style. Nat. Rev. Mol. Cell Bio..

[99] Poss KD (2010). Advances in understanding tissue regenerative capacity and mechanisms in animals. Nat. Rev. Genet..

[100] Mendez-Ferrer S (2010). Mesenchymal and haematopoietic stem cells form a unique bone marrow niche. Nature.

[101] Tan JM (2012). Induction therapy with autologous mesenchymal stem cells in living-related kidney transplants a randomized controlled trial. JAMA.

[102] Sensebe, L., Gadelorge, M. & Fleury-Cappellesso, S. Production of mesenchymal stromal/stem cells according to good manufacturing practices: a review. *Stem Cell Res. Ther.* **4**, 66 (2013). 10.1186/scrt217 PMC3707032 23751270

[103] Presen, D. M., Traweger, A., Gimona, M. & Redl, H. Mesenchymal stromal cell-based bone regeneration therapies: from cell transplantation and tissue engineering to therapeutic secretomes and extracellular vesicles. *Front. Bioeng. Biotechnol.* **7**, 352 (2019). 10.3389/fbioe.2019.00352 PMC6890555 31828066

[104] Confalonieri D, Schwab A, Walles H, Ehlicke F (2018). Advanced therapy medicinal products: a guide for bone marrow-derived MSC application in bone and cartilage tissue engineering. Tissue Eng. Part B-Rev..

[105] Khademhosseini A, Langer R (2016). A decade of progress in tissue engineering. Nat. Protoc..

[106] Pace LA, Plate JF, Smith TL, Van Dyke ME (2013). The effect of human hair keratin hydrogel on early cellular response to sciatic nerve injury in a rat model. Biomaterials.

[107] Rosenzweig DH (2012). Culture of primary bovine chondrocytes on a continuously expanding surface inhibits dedifferentiation. Tissue Eng. Part A.

[108] Rosenzweig DH, Solar-Cafaggi S, Quinn TM (2012). Functionalization of dynamic culture surfaces with a cartilage extracellular matrix extract enhances chondrocyte phenotype against dedifferentiation. Acta Biomater..

[109] Pei M, He F (2012). Extracellular matrix deposited by synovium-derived stem cells delays replicative senescent chondrocyte dedifferentiation and enhances redifferentiation. J. Cell Physiol..

[110] Yang YH (2018). Mesenchymal stem cell-derived extracellular matrix enhances chondrogenic phenotype of and cartilage formation by encapsulated chondrocytes in vitro and in vivo. Acta Biomater..

[111] Pahoff S (2019). Effect of gelatin source and photoinitiator type on chondrocyte redifferentiation in gelatin methacryloyl-based tissue-engineered cartilage constructs. J. Mater. Chem. B.

[112] Ozturk E (2016). Sulfated hydrogel matrices direct mitogenicity and maintenance of chondrocyte phenotype through activation of FGF signaling. Adv. Funct. Mater..

[113] Jin, G. Z. & Kim, H. W. Efficacy of collagen and alginate hydrogels for the prevention of rat chondrocyte dedifferentiation. *J. Tissue Eng.* **9**, 2041731418802438 (2018). 10.1177/2041731418802438 PMC6176533 30305887

[114] Li SY (2015). Effects of nanoscale spatial arrangement of arginine-glycine-aspartate peptides on dedifferentiation of chondrocytes. Nano Lett..

[115] Chattopadhyay S, Shubayev VI (2009). MMP-9 controls Schwann cell proliferation and phenotypic remodeling via IGF-1 and ErbB receptor-mediated activation of MEK/ERK pathway. Glia.

[116] Liu H (2010). Matrix metalloproteinase inhibition enhances the rate of nerve regeneration in vivo by promoting dedifferentiation and mitosis of supporting schwann cells. J. Neuropathol. Exp. Neurol..

[117] Rittchen S (2015). Myelin repair in vivo is increased by targeting oligodendrocyte precursor cells with nanoparticles encapsulating leukaemia inhibitory factor (LIF). Biomaterials.

[118] Xu C (2011). Recombinant human midkine stimulates proliferation and decreases dedifferentiation of auricular chondrocytes in vitro. Exp. Biol. Med..

[119] Luo LK, Wei QJ, Liu L, Zheng L, Zhao JM (2015). Andrographolide enhances proliferation and prevents dedifferentiation of rabbit articular chondrocytes: an in vitro study. Evid. Based Complement Alternat. Med..

[120] Sunico CR, Portillo F, Gonzalez-Forero D, Kasparov S, Moreno-Lopez B (2008). Evidence for a detrimental role of nitric oxide synthesized by endothelial nitric oxide synthase after peripheral nerve injury. Neuroscience.

[121] Sunico CR, Moreno-Lopez B (2010). Evidence for endothelial nitric oxide as a negative regulator of Schwann cell dedifferentiation after peripheral nerve injury. Neurosci. Lett..

[122] Santangelo KS, Nuovo GJ, Bertone AL (2012). In vivo reduction or blockade of interleukin-1beta in primary osteoarthritis influences expression of mediators implicated in pathogenesis. Osteoarthr. Cartil..

[123] Fujino M (2003). CrmA gene expression protects mice against concanavalin-A-induced hepatitis by inhibiting IL-18 secretion and hepatocyte apoptosis. Gene Ther..

[124] Ma BL (2015). Inhibition of interleukin-1beta-stimulated dedifferentiation of chondrocytes via controlled release of CrmA from hyaluronic acid-chitosan microspheres. BMC Musculoskelet. Disord..

[125] Cha BH, Lee JS, Kim SW, Cha HJ, Lee SH (2013). The modulation of the oxidative stress response in chondrocytes by Wip1 and its effect on senescence and dedifferentiation during in vitro expansion. Biomaterials.

[126] D’uva G (2015). ERBB2 triggers mammalian heart regeneration by promoting cardiorlyocyte dedifferentiation and proliferation. Nat. Cell Biol..

[127] Liu Y (2011). Dedifferentiation-reprogrammed mesenchymal stem cells with improved therapeutic potential. Stem Cells.

[128] Liu Y (2010). Switching from bone marrow-derived neurons to epithelial cells through dedifferentiation and translineage redifferentiation. Cell Biol. Int..

[129] Lin S (2017). Stepwise preconditioning enhances mesenchymal stem cell-based cartilage regeneration through epigenetic modification. Osteoarthr. Cartil..

[130] Duan L (2017). DNA methylation profiling in chondrocyte dedifferentiation in vitro. J. Cell Physiol..

[131] Hong EH (2014). Low-dose gamma-radiation inhibits IL-1beta-induced dedifferentiation and inflammation of articular chondrocytes via blockage of catenin signaling. IUBMB Life.

[132] Jimenez G (2015). Activin A/BMP2 chimera AB235 drives efficient redifferentiation of long term cultured autologous chondrocytes. Sci. Rep..

[133] Yao Y (2011). In vitro study of chondrocyte redifferentiation with lentiviral vector-mediated transgenic TGF-beta3 and shRNA suppressing type I collagen in three-dimensional culture. J. Tissue Eng. Regen. Med..

[134] Schrobback K (2011). Adult human articular chondrocytes in a microcarrier-based culture system: expansion and redifferentiation. J. Orthop. Res..

[135] Zeng L (2015). Redifferentiation of dedifferentiated chondrocytes in a novel three-dimensional microcavitary hydrogel. J. Biomed. Mater. Res. A.

[136] Schrobback K (2012). Effects of oxygen and culture system on in vitro propagation and redifferentiation of osteoarthritic human articular chondrocytes. Cell Tissue Res..

[137] Babur BK (2013). The interplay between chondrocyte redifferentiation pellet size and oxygen concentration. PLoS ONE.

[138] Levett PA (2014). Chondrocyte redifferentiation and construct mechanical property development in single-component photocrosslinkable hydrogels. J. Biomed. Mater. Res. A.

